# Confirmatory Factor Analysis of WAIS-IV in a Clinical Sample: Examining a Bi-Factor Model

**DOI:** 10.3390/jintelligence5010002

**Published:** 2016-12-30

**Authors:** Rachel Collinson, Stephen Evans, Miranda Wheeler, Don Brechin, Jenna Moffitt, Geoff Hill, Steven Muncer

**Affiliations:** 1 Neuropsychology Department, James Cook University Hospital, South Tees Hospitals NHS Foundation Trust, Middlesbrough TS4 3BW, UK; r.collinson@nhs.net (R.C.); Stephen.Evans@stees.nhs.uk (S.E.); Miranda.Wheeler@stees.nhs.uk (M.W.); Donald.Brechin@stees.nhs.uk (D.B.); Jenna.Moffitt@stees.nhs.uk (J.M.); Geoffrey.Hill@stees.nhs.uk (G.H.); 2 Clinical Psychology, Teesside University, Middlesbrough TS1 3BX, UK

**Keywords:** WAIS, confirmatory factor analysis, bi-factor model, clinical sample

## Abstract

There have been a number of studies that have examined the factor structure of the Wechsler Adult Intelligence Scale IV (WAIS-IV) using the standardization sample. In this study, we investigate its factor structure on a clinical neuropsychology sample of mixed aetiology. Correlated factor, higher-order and bi-factor models are all tested. Overall, the results suggest that the WAIS-IV will be suitable for use with this population.

## 1. Introduction

The Wechsler Adult Intelligence Scale-IV (WAIS-IV) [1] incorporates significant structural and content revisions in comparison to the WAIS-III [2]. The complete WAIS-IV comprises 15 subtests, 10 of which are core subtests required to compute the Full Scale IQ (FSIQ), and four Index Scores that measure specific domains of cognitive functioning. The WAIS-IV manual outlines the underlying four-factor structure of the WAIS-IV [1], where there is strong construct validity support for the four indexes, comprising: Verbal Comprehension (VC); Perceptual Reasoning (PR); Working Memory (WM); and, Processing Speed (PS). Each of these Indexes contributes to providing a composite score that represents overall general intellectual ability (i.e. Full Scale IQ). The four-factor model contributes to an overall second-order g factor, known as the Full Scale IQ, which comprises overall general intelligence. 

In the last few years, a number of studies have investigated what has been called the bi-factor [3] or direct hierarchical model [4] of intelligence. For this model, all of the latent variables in the model are defined by the subtests directly. This means that the general factor is a first-order factor in the bi-factor model, and not a higher-order factor defined by the first-order factors. The bi-factor model has been shown to be a better fit to WAIS-IV data collected from a clinical sample attending a university-based clinic specializing in learning disabilities [5]. In fact, the bi-factor model fit was similar to that of a four correlated factors model, comprising the four indexes above.

Recently, the bi-factor approach has been criticised for an inherent statistical bias that makes it more likely to have acceptable fit statistics than the higher-order factor model [6]. Furthermore, Morgan, Hodge, Wells and Watkins [7] provided some support for such a bias in a simulation study that found that, “when samples were generated from a true higher-order factor structure, approximate fit statistics tended to identify the bi-factor solution as best fitting.” More recently, Gignac [8] has tried to explain the bias in terms of the proportionality constraint [9]. The higher-order factor model requires the general factor and specific factor loadings to be equal, whereas the bi-factor model does not contain this constraint. Gignac [8] demonstrated this effect in a series of simulations in which the correlations between subtests became increasingly unequal so that the general-to-specific ratio became proportionately larger. The fit statistics of the higher-order model became progressively worse than the bi-factor model. It has also been suggested that the Tucker Lewis Index (TLI), the Akaike Information Criterion (AIC) and the Bayesian Information Criterion (BIC), which contain a penalty for model complexity, should be used when comparing higher-order and bi-factor models [10]; however, it should be noted that the very nature of these indices works in favour of a bi-factor model. 

In the present study, we compare possible factor structures of the WAIS-IV in a mixed clinical sample of patients attending a Neuropsychological Service of a large British hospital. This is important as it has been acknowledged that data from clinical samples could produce different results from the many studies that have analysed standardization data [11], and there are relatively few studies that have used clinical samples [5,12]. Nelson et al. [5] examined the fit of four first-order factor models, a hierarchical and a bi-factor model for data from a university-based learning disorders clinic. Weiss et al. [12] looked at four- and five-factor solutions for the WAIS-IV clinical sample of 411 participants. Specifically, we compare one, two, three and four first-order factor models, as well as the bi-factor and higher-order factor models, and also use the fit indices that have been recommended for such comparisons [10]. 

## 2. Method 

### 2.1. Participants

The clinical sample was recruited from an anonymised database of neuropsychology patients with mixed aetiology, who had attended for neuropsychological assessment. Patients were selected from the database if they fulfilled the following inclusion criteria:

(a) All three Perceptual Reasoning Index subtests from the WAIS-IV (i.e. Block Design, Matrix Reasoning, and Visual Puzzles (VP)) were administered. This requirement was due to the data being collected as part of a broader project looking into the validity and reliability of the new Visual Puzzles subtest in the WAIS battery.
(b) Predicted pre-morbid Full Scale IQ was over 70, as determined by the Test of Premorbid Functioning (TOPF) [13].

Based on the inclusion criteria, the total clinical sample comprised 161 participants (males, *N* = 94; females, *N* = 67). The average age of the clinical sample was 52.08 years (SD = 14.29) and ranged from 20 to 82 years. Table 1 outlines a breakdown of the various diagnoses within the sample.

### 2.2. Instruments

#### Wechsler Adult Intelligence Scale—Fourth Edition (WAIS-IV) [1]

The WAIS-IV is a neuropsychological measure of cognitive ability in adults and includes updated normative data for ages 16–90 years. The WAIS-IV consists of 10 core subtests (Vocabulary, Information, Similarities, Digit Span, Arithmetic, Block Design, Matrix Reasoning, Visual Puzzles, Coding, and Symbol Search), and five supplemental subtests (Letter-Number Sequencing, Figure Weights, Comprehension, Cancellation, and Picture Completion), with the 10 core subtests comprising the Full Scale IQ. There are four index scores representing major components of intelligence: Verbal Comprehension Index (VCI); Perceptual Reasoning Index (PRI); Working Memory Index (WMI); and, Processing Speed Index (PSI). Two broad scores are also generated, which can be used to summarise general intellectual abilities:

- Full Scale IQ (FSIQ), based on the total combined performance of the VCI, PRI, WMI, and PSI.
- General Ability Index (GAI), based only on the six subtests that comprise the VCI and PRI.

The reliability and validity of the WAIS-IV is outlined in the Technical and Interpretive Manual [1]). The main reliability figure based on the standardisation sample for Full Scale IQ was 0.98, indicating good internal consistency. In addition, the test-retest reliability of 0.96 was found for Full Scale IQ when 298 participants were given the assessment twice with a mean interval of 22 days. In regard to validity, factor analytic studies [1] demonstrate the WAIS-IV to be a good measure of general intelligence (*g*) and of the cognitive abilities measured in the index scales.

### 2.3. Data Analysis

All models were fit using the lavaan package designed by Rosseel, [14] which is available in R. Maximum likelihood estimation was used for all models. The models that were tested were the same as Nelson et al. [5]; a one-factor, two oblique verbal and nonverbal factors; three oblique verbal, nonverbal and combined working memory/processing speed factors; a four oblique verbal, nonverbal, working memory and processing speed factors; a higher-order model with four correlated first-order factors and a bi-factor model.

Nelson et al. [5] used the comparative fit index (CFI), root mean square error of approximation (RMSEA), chi-square and AIC as fit measures. We have also included the TLI and BIC. As some of the models are nested, the difference in chi square and other fit indices can be used for model comparison.

## 3. Results

Model fit statistics are presented in Table 2. It is clear that the one, two and three factor solutions are inadequate. The four-factor model has a CFI, RMSEA and TLI which indicate reasonable fit [15]. The correlated four-factor model was also a better fit than the higher-order factor model (ΔΧ^2^ = 8.33, *p* < 0.05). The model with the best fit, however, appears to be the bi-factor model, which has the highest TLI and the lowest AIC, BIC and RMSEA. The factor loadings for the four correlated factors model, the bi-factor model and hierarchical model are given in Table 3. The difference in TLI between the bi-factor and higher-order factor model is 0.022, which is higher than the suggested threshold for indication of a practical improvement in models of 0.01 [10]. The difference in BIC of 13.85 is also higher than the suggested threshold of 10. The bi-factor model is also a better fit than the four correlated factors model. It should be noted, however, that for this model it is necessary to constrain two indicators of the third and fourth factors to be equal to ensure identification, as Nelson et al. [5] did. 

The variance accounted for by the different elements of the bi-factor model was also estimated. The general factor accounted for 69.34% of the common variance. The VC factor accounted for 11.21% of the common variance, the PR factor for 8.33%, the WM factor for 2.08% and the PS factor 9.04%. Lastly, the hierarchical omega, which estimates the latent construct reliability, was also estimated. The general factor ω_h_ was 0.84, VC = 0.33, PR = 0.20, WM = 0.08, and PS = 0.31. The general factor appears to be even more important in the current data than it was in Nelson et al. [5], where it accounted for 52.5% of common variance.

## 4. Discussion

The current study finds similar results to those reported by Nelson et al. [5], although less variance is accounted for by the specific factors. The WM factor in particular is substantially lower in this study, and Arithmetic in particular has a low specific loading onto it. Overall, the pattern of loading and low omega of the WM factor suggest it is mostly measuring *g*. This is something that should be looked at in future research with clinical samples. The four-factor solution was both the best and only one of the first-order factor models to have satisfactory fit statistics. The higher-order factor model had reasonable, but not as good, fit statistics as the correlated factor model. We also replicated the finding of a better fit for the bi-factor model and found that the bi-factor model was better than the correlated factor model. 

It should be noted that the pattern of correlations between the index variables was such that it was clear that the proportionality constraint would have been broken [8]. Therefore, perhaps the most important finding from this study is that a four-factor structure seems to adequately fit the data from a clinical sample. 

We conclude that the WAIS-IV provides robust evidence for the measurement of general intelligence in a mixed clinical sample, as has been previously found in non-clinical samples. The data also provide evidence for a four-factor structure, which is useful information for practicing psychologists and neuropsychologists, as it indicates that the model as proposed in the WAIS-IV technical manual [1] can been replicated in a large clinical sample of mixed aetiology.

## Figures and Tables

**Table 1:** Diagnoses Total Sample Percentages.

Diagnosis	*N*	%
Aneurysm	5	3.11
Brain dysfunction	25	15.53
Dementia	21	13.04
Epilepsy	13	8.07
Head injury	17	10.56
Multiple sclerosis	8	4.97
Other	34	21.12
Parkinson’s	8	4.97
Subarachnoid haemorrhage	15	9.32
Tumour	20	12.42

**Table 2:** Confirmatory Factor Analysis Fit Statistics for WAIS –IV.

Model	×2	df	CFI	TLI	AIC	BIC	RMSEA	90%CI	TLI DIFF	BIC DIFF	RMSEA DIFF
One factor	164.51	35	0.838	0.791	7437.59	7436.02	0.151	0.175			
Two factors	121.28	34	0.891	0.855	7396.35	7394..71	0.126	0.15	0.064	41.31	0.025
Three factors	80.07	32	0.94	0.915	7359.14	7357.34	0.096	0.123	0.06	37.37	0.03
Four factors	50.38	29	0.973	0.958+.	7335.45	7333.42	0.067	0.098	0.043	23.92	0.029
Hierarchical Models											
Higher order	58.71	31	0.965	0.95	7339.78	7337.91	0.074	0.103	−0.008	−4.49	−0.007
Bi-factor	37.18	27	0.987	0.979	7326.25	7324.06	0.048	0.083	0.022	13.85	0.026

Note: CFI = comparative fit index; TLI = Tucker-Lewis Index; AIC = Akaike Information Criterion; BIC = Bayesian Information Criterion; RMSEA = root mean square error of approximation; CI = confidence interval; DIFF = difference.

**Table 3:** Standardised factor loadings for correlated factors, bi-factor and hierarchical models.

Correlated Model			Bi-factor Model					Hierarchical Model				
Subtest	Factor	Load	Subtest	Factor	Load	Factor	Load	Subtest	Factor	Load	Factor	Load
Si	VC	0.72	Si	VC	0.37	G	0.61	Si	VC	0.73	G	0.74
Vo	VC	0.82	Vo	VC	0.53	G	0.61	Vo	VC	0.81	“	“
Inf	VC	0.69	Inf	VC	0.55	G	0.49	Inf	VC	0.7	“	“
BD	PR	0.8	BD	PR	0.5	G	0.66	BD	PR	0.8	G	0.88
MR	PR	0.67	MR	PR	0.08*	G	0.69	MR	PR	0.68	“	“
VP	PR	0.75	VP	PR	0.53	G	0.6	VP	PR	0.74	“	“
DS	WM	0.76	DS	WM	0.31	G	0.76	DS	WM	0.83	G	0.95
Ar	WM	0.8	Ar	WM	0.19	G	0.8	Ar	WM	0.82	“	“
SS	PS	0.68	SS	PS	0.64	G	0.68	SS	PS	0.8	G	0.82
Cd	PS	0.74	Cd	PS	0.41	G	0.74	Cd	PS	0.91	“	“

Notes: Subtest; Si = Similarities; Vo = Vocabulary; Inf = Information; BD = Block Design; MR = Matrix Reasoning; VP = Visual Puzzles; DS = Digit Span; Ar = Arithmetic; SS = Symbol Search; Cd = Coding. Factor; VC = Verbal Comprehension; PR = Perceptual Reasoning; WM = Working Memory; PS = Processing Speed. Load = standardized factor loading, all loadings significant at <0.05, except for Bi-factor Model loading MR on PR*.
